# Cancer patients’ behaviors and attitudes toward natural health products

**DOI:** 10.1186/s12906-023-04278-0

**Published:** 2023-12-06

**Authors:** Audrey Schils, Anne-Sophie Lechon, Sarah Rondeaux, Florence Souard, Jean-Luc Van Laethem, Stephanie Pochet, Veronique Mathieu, Carine De Vriese

**Affiliations:** 1 Department of Pharmacotherapy and Pharmaceutics, Faculté de Pharmacie, Université Libre de Bruxelles (ULB), Boulevard du Triomphe 9040, 1050 Brussels, Belgium; 2 Department of Gastroenterology and Digestive Oncology, Erasme University Hospital, Université Libre de Bruxelles (ULB), Route de Lennik 808, 1070 Brussels, Belgium

**Keywords:** Complementary and alternative medicine, Natural health products, Cancer, Behaviors, Attitudes, Descriptive study, Integrative oncology, Online questionnaire, Belgium

## Abstract

**Background:**

Natural health products (NHPs), including vitamins, minerals, and herbal supplements, are the most common complementary and alternative medicine (CAM) among cancer patients. Our survey determined the attitudes and behaviors of cancer patients toward natural complementary therapies that should be considered to implement an integrative approach in the future.

**Methods:**

Our survey was conducted in four hospitals in Belgium. Questionnaires were posted online from October 2020 to October 2021 for cancer patients. Descriptive statistics were used to analyze the data. A $\chi ^{2}$ test was applied to study the type of NHP consumed according to diagnosis time. Fischer’s exact test compared patients who had changed their consumption since diagnosis and those who had not.

**Results:**

Out of 349 questionnaires collected, only 59 met all inclusion criteria. 83.1 % of the patients agreed that conventional medicine (CM) could benefit from complementary therapies, but they did not estimate (72.3 % of the patients) that those latter are more effective than conventional medicine. More than half of the patients used five or more NHPs. The most frequent NHPs consumed daily were vitamins (64.4 %), followed by other products (i.e., probiotics, gemmotherapy, birch sap and omega 3/6) (42.4 %) and herbs (40.7 %). Almost all patients started taking NHPs before their cancer diagnosis, but 72.7 % have changed their consumption significantly (*p* = 0.009) since their diagnosis. Boosting the immune system (79.7 %) and limiting conventional treatment side effects (76.9 %) were the most common reasons for NHPs’ use. 74.4 % of the patients did not take complementary therapies to delay or avoid conventional treatment.

**Conclusions:**

The combination and high diversity of NHPs consumption highlight the importance of educating patients and healthcare providers (HCPs) about the risk of drug interactions associated with these natural products. Most cancer patients are more interested in using this non-mainstream medicine to complement their conventional treatment than as an alternative. Knowing the patients' reasons and understanding patients’ attitudes toward NHPs will be essential for HCPs to address NHPs’ use.

**Supplementary Information:**

The online version contains supplementary material available at 10.1186/s12906-023-04278-0.

## Background

Complementary and alternative medicine (CAM), according to the National Center for Complementary and Integrative Health (NCCIH), includes an extensive range of products and different medical practices. Even if these non-mainstream approaches are often used interchangeably, the terms refer to two concepts. Complementary medicine refers to CAM used together with conventional medicine. Alternative medicine, on the other hand, is used instead of standard treatment. CAM therapies are classified into four categories:

1. Nutritional approaches or natural health products (NHPs) (e.g., dietary supplements, herbs, probiotics, vitamins);
2. Psychological approaches (e.g., meditation, hypnosis);
3. Physical approaches (e.g., massage, acupuncture);
4. Other complementary approaches (e.g., homeopathy, naturopathy) [1].

Over the years, CAM use has increased dramatically, especially among cancer patients, ranging from 10 % to 76 % worldwide [2–4]. In particular, several studies have shown that CAM therapies use increases after cancer diagnosis [5–7]. Buckner et al. [3] showed that NHPs use was three times lower before diagnosis than after diagnosis. Despite this, Luo et al. [8] did not find a substantial difference between the consumption of these natural supplements before (71 %) and after cancer treatment (69 %). Therefore, evaluating how a cancer diagnosis can influence NHPs’ consumption appears challenging. Even determining the exact prevalence of CAM use by cancer patients is difficult mainly because of the lack of consensus on the CAM definition [9]. Indeed, some surveys include only herbal medicines in the definition of CAM, while others take into account all non-mainstream medical practices (e.g., massage, acupuncture) and dietary supplements [5, 10, 11]. Reaching a consensus on the definition of CAM is further complicated by cultural differences regarding the definition of mainstream medicine. Furthermore, the sample size, the data collection method, and the characteristics of the population differ among studies. They also contribute to the lack of reliability in CAM consumption data [9].

It’s important to note that NHPs are subject to complex legislation, with different statuses depending on their function, composition, and presentation. One common way NHPs are marketed is as dietary supplements. The European Union has established a framework directive (2002/46/EC) to start unifying national laws for dietary supplements [12]. In Belgium, dietary supplements are defined as foods in pre-dosed form (capsules, lozenges, droppers, etc.) containing nutrients, plants, or other substances with a nutritional or physiological effect. These supplements are regulated by three specific royal decrees [13–15]. The one concerning nutrients, i.e., vitamins and minerals, outlines the minimum and maximum levels per daily portion and requirements regarding labeling and advertising. The legislation governing plants specifies a list of prohibited and authorized plants, sometimes with a defined maximum content limit, such as in the case of St. John’s wort, where the daily portion cannot exceed 700 $\upmu$g of hypericin. Moreover, dietary supplements are not subject to the same rigorous regulatory standards as drugs. They do not have to meet the same manufacturing and quality control standards or prove their efficacy or safety before marketing. They also cannot claim to prevent or cure medical conditions or diseases. Another issue with dietary supplements is the lack of post-launch safety and effectiveness assessment, which is mandatory for drugs. Although drugs and dietary supplements may appear similar, their regulatory differences are significant. It is crucial to inform patients of these differences [16, 17].

NHPs, including vitamins, minerals, and herbal remedies, are used mainly by cancer patients [6, 18, 19] as well as homeopathy [20, 21]. Currently, there is only limited evidence about the safety and efficacy of these natural supplements [22]. The main concern surrounding NHPs use is the risk of drug interactions with conventional treatment. This can lead to adverse reactions (ARs) or lower treatment efficacy [23, 24]. Firkins et al. [25] found a risk of interaction between NHPs and conventional treatment for 54.9 % of the CAM users. For example, garlic decreases docetaxel clearance and may increase its adverse reactions due to accumulation. On the other hand, St John’s wort, a strong CYP3A4 inducer, significantly decreases docetaxel plasma concentration and may reduce treatment efficacy [26]. This interaction risk is difficult to evaluate for the following reasons:

1. Lack of *in vivo* data and clinical studies investigating CAM substances interactions. *In vitro* studies and preclinical animal studies are used to extrapolate most interactions. Only a few studies in the human population with accurate reporting confirm this level of risk [26–29].
2. These supplements can contain several biologically active components (e.g., supplements of vitamins containing a mixture of antioxidants) compared to conventional medicines’ single active compounds [19, 29].
3. Difficulties evaluating the exact substances the patient takes, dosage remains undefined, and data on time between chemotherapy and NHPs lack [24].

However, patients might perceive NHPs as “safe” according to their “natural” origin compared to conventional medicine, despite not being harmless in practice [7, 30]. Since they estimate NHPs as “safe”, they generally do not communicate their use of CAM to their healthcare providers (HCPs). Moreover, patients sometimes fear that HCPs may perceive their uses as disrespectful, which prevents them from disclosing NHPs’ uses [22, 31]. Indeed, the non-disclosure rate is high and estimated at 80 % [8]. In addition, the Internet and social networks provide many easily accessible information to the patients [32]. Consequently, self-diagnosis and self-treatment are increasing. Despite highly accessible information, patients encounter difficulties identifying evidence-based sources [29]. Furthermore, personal networks, such as friends, family and the media, are an invaluable non-evidence-based source of information about CAM [33]. Philak et al. [34] found that 82 % of the patients fully trusted the information received, regardless of the source.

Most cancer patients are more interested in using these non-mainstream medicines to complement their conventional treatment than as an alternative [35–38]. Indeed, boosting the immune system, improving well-being, reducing conventional treatment side effects and playing an active role in their treatment are the most frequent reasons for CAM use cited by cancer patients [33, 39] . However, patients’ beliefs should not be forgotten. Indeed, attitudes toward these non-mainstream therapies may also influence the decision to seek CAM supplements. Though few studies explore the two factors simultaneously, this knowledge could be helpful in modifying conventional treatment by incorporating some aspects of CAM [40].

For all the reasons cited above, drawing conclusions about patients’ attitudes and behaviors toward complementary therapies, and evaluating how the diagnosis impacts their use, is a complicated task. To better understand why cancer patients use complementary therapies, it is essential to investigate consumption across different countries and cultures [18]. Our survey aimed to study the attitudes and behaviors of cancer patients toward natural complementary therapies among a sample of French-speaking patients in Belgium. The types of NHPs consumed included vitamins, minerals, gemmotherapy, probiotics, omega 3/6, birch sap, homeopathy, Bach flowers (BFs), Essential oils (EOs), herbal teas and plants.

## Method

### Questionnaire content

The study was carried out using an online questionnaire of 22 questions divided into four main parts:

1. Participants’ characteristics;
2. Attitudes toward complementary therapies and conventional medicine;
3. Habits and reasons for NHPs use;
4. Sources of information and expectations of NHPs.

This survey used nine questions from McFadden et al.’s Complementary, Alternative and Conventional Medicine Attitudes Scale (CACMAS) [40] to measure attitudes toward complementary therapies and conventional medicine. CACMAS was created to understand how the attitudes of healthcare recipients affect CAM use. The other questionnaire sections were developed using literature [3, 20, 41, 42]. The questionnaire was translated into French. Different reviewers validated the questionnaire layout for understanding, content, relevance, and logic. Among all these questions, five assessed the participants’ characteristics, nine were about patients’ attitudes toward complementary therapies and conventional medicine, questions were related to patients’ behaviors toward NHPs, and the source of information was the last item measured. The answer options were multiple/single choice, dichotomous, short open-ended box and 11-Likert or 5-Likert scale from “strongly disagree” to “strongly agree”. An additional file shows the questionnaire in details (Additional file 1).

### Data collection and procedures

The questionnaire was first posted online from October 2020 to January 2021. The survey was extended to October 2021 due to COVID-19 global pandemic slowing the data collection. Patients could access the questionnaire for one year by scanning a QR code or following a link printed on flyers and posters. Data were collected from the Jules Bordet Institute, the Erasme Hospital (ULB), the Centre Hospitalier Régional (CHR) de la Haute Senne and the CHIREC Hospital Group. The distribution method of the questionnaire was specific to each hospital. Posters were put up and flyers were available in waiting rooms, at the hospitals pharmacy, or distributed by nurses directly to patients. The survey was also shared on social networks (i.e., Facebook, Instagram, and LinkedIn). All these recruitment methods resulted in a snowball sampling of the data.

### Participants and inclusion criteria

Patients were eligible to complete the questionnaire if they met the following criteria:

- They were French-speaking adult patients;
- The questionnaire had to be completed by the patient himself or a relative in the patient’s presence;
- They were receiving an anticancer treatment [antiblastic chemotherapy (L01 ATC) and/or hormone antagonists (L02B ATC)] at the time of the survey or had received an anticancer treatment no more than five years before participating in the survey;
- They consented to participate in the study;
- They answered the part of the questionnaire “habits and reasons for NHPs’ use” (cf. point 3 of the questionnaire content).

**Fig. 1:**
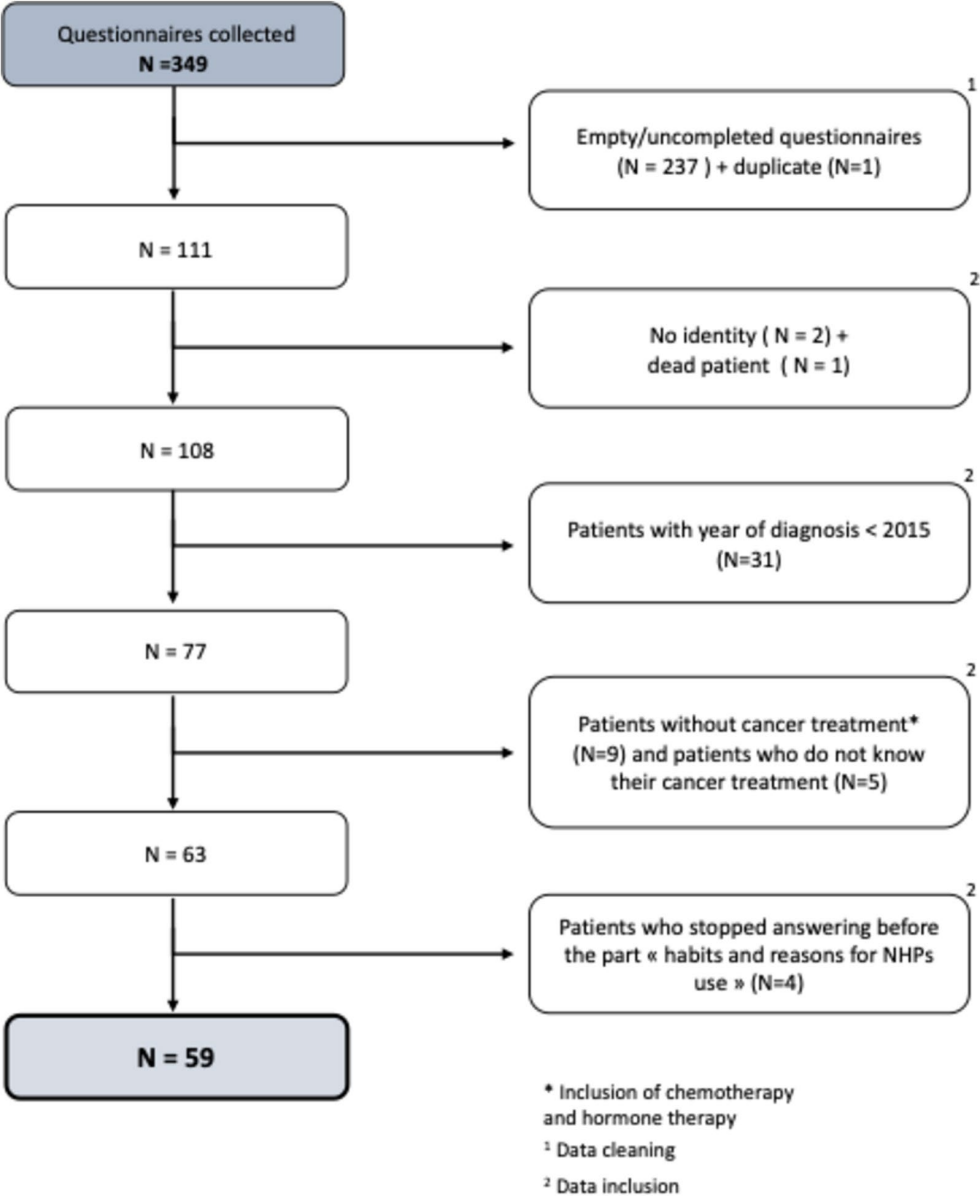
Data cleaning and inclusion process

Patients who did not meet the inclusion criteria were excluded from the analysis. The data cleaning and inclusion process are presented in Fig. 1.

### Statistics and data analysis

Descriptive statistics (frequencies and percentages) were conducted using Excel^®^ version 16.66.1. The open-ended questions were first coded into categorical variables. The results were analyzed for the questions with 5-Likert and 11-Likert scales by grouping the answers into three categories; 1) disagree, 2) agree and 3) neutral. To investigate the relationship between diagnosis time and NHP type used, the $\chi ^{2}$ test of independence was applied. Fischer’s exact test was used to determine if there was a significant difference between patients who had changed their consumption since diagnosis and those who had not. These statistical analyzes were performed with R studio ^®^ version 2022.07.1.

The eleven different NHPs were grouped into six families:

1. Vitamins;
2. Bach flowers (BFs) and homeopathy;
3. Minerals;
4. Essential oils (EOs);
5. Herbs;
6. Other products.

To facilitate analysis, our classification process was based on similarities. Since homeopathy and BFs are based on dilution, they were combined. The “other products” category included gemmotherapy, probiotics, birch sap and omega 3/6. Plants and herbal teas belong to the category of herbs. Merging helped to increase the number of responses in each family and removed the risk of misclassifying similar products. Unfortunately, the categorization could not solely rely upon Belgian legislation. For instance, essential oils can be marketed as medicinal products, dietary supplements, or cosmetics, while herbal products can be marketed as either food supplements or medicines. Classifying certain items, like Bach flowers, can be particularly complex, and a commission is usually required to make the ultimate decision. These examples demonstrate the complexity of product classification based on legislation.

The sources of information have been divided into three categories:

1. Healthcare providers;
2. Alternative HCPs;
3. Other sources.

Combining the sources made it possible to determine whether the main information source was medical. Healthcare providers included oncologists, physiotherapists, nurses, pharmacists, and general practitioners (GPs). Homeopaths and naturopaths were grouped into another category called alternative HCPs. Media, family, friends, and their close circle were listed as other sources.

Table 1 illustrates how the merging of NHPs influenced how questions were analyzed regarding behaviors.

**Table 1:** An example explaining how NHPs fusion influences the analysis. Patient X uses homeopathy & BFs

Question topic	Problematic answer	Hypothesis	Selection method	Selected answer
Type & frequency?	H^a^: daily	What is the most used NHP?	Highest frequency	Daily consumption of H & BFs
	BFs: monthly			
Started using?	H: before the diagnosis	Did the diagnosis affect consumption?	Closest time to the diagnosis	Use of H & BFs since the diagnosis
	BFs: at the diagnosis			
Experience?	H: adverse reaction	Is there an adverse reaction or a beneficial effect?	Mean^b^	AR after intake of H & BFs
	BFs: no effect			

^a^H stands for homeopathy

^b^For the question about experience, we checked that the same patient did not report adverse reaction and beneficial effect from taking NHPs in the same category

## Results

### Participants’ characteristics

Out of 349 questionnaires collected, 59 were selected after data cleaning and excluding patients who did not meet the inclusion criteria (Fig. 1).

Of the 59 questionnaires selected, 68 % (*n* = 40) were completed, while 32 % (*n* = 19) were partially filled. The patient characteristics are summarized in Table 2. Among the participants, more than half were women 55.9 % (*n* = 33). For 61 % ( *n* = 36) of the participants, the diagnosis dated from the past two years. Finally, 63.5 % (*n*= 47) of the patients took antineoplastic agents and 36.5 % (*n* = 27) took hormone antagonists and related treatments, including gonadorelin inhibitors and antiandrogens.

**Table 2:** Patients’ characteristics

Patients’ characteristics	n	%
*Age in years*		
22-41	8	13.6%
42-61	19	32.2%
62-81	13	22.0%
Unknown	19	32.2%
*Gender*		
Women	33	55.9%
Men	7	11.9%
Unknown	19	32.2%
*Country of the treatment*		
Belgium	38	64.4%
France	1	1.7%
Switzerland	1	1.7%
Unknown	19	32.2%
*Year of diagnosis*		
6 years ago	3	5.1%
5 years ago	5	8.5%
4 years ago	8	13.6%
3 years ago	7	11.9%
$\le$ 2 years ago	18	30.5%
*Type of cancer treatment*		
Antineoplastic agents	47	63.5%
Hormone antagonists & related treatments	7	36.5%

### Attitudes toward complementary therapies

**Fig. 2:**
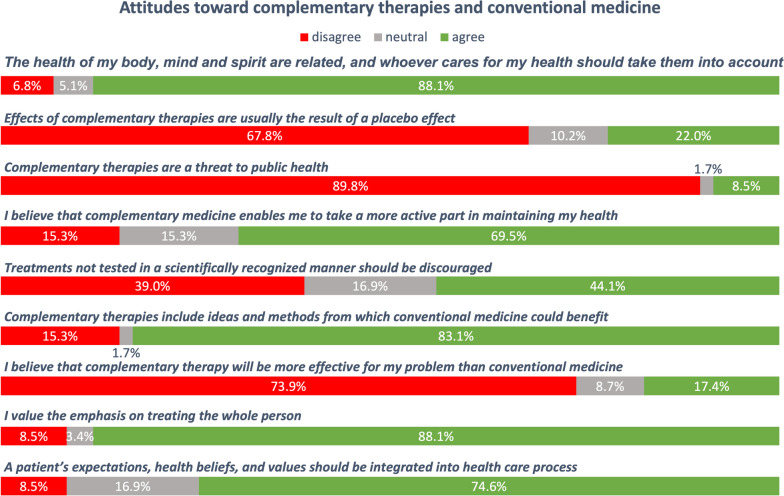
Attitudes toward complementary therapies and conventional medicine

Figure 2 illustrates patients’ attitudes toward complementary therapy and conventional medicine. Most patients agreed with the following items:

- *The health of my body, mind and spirit are related, whoever cares for my health should take them into account* (88.1 %, *n* = 52).
- *Complementary therapies include ideas and methods from which CM could benefit* (83.1 %, *n* = 49)
- *I value the emphasis on treating the whole person * (88.1 %, *n* = 52).

Patients did not perceive complementary therapies as a threat to public health (89.9 %, *n* = 53) or more effective than conventional medicine (73.9 %, *n* = 34).

### Behavior toward NHPs

A minimum of five different NHPs were consumed by 52.5 % (n = 31) of the patients. Vitamins (86.4 %, *n* = 51) and herbs (84.7 %, *n* = 50) were the two most consumed supplements (Table 3). Other products are the third most widely used supplements (69.5 %, *n* = 41). Among the 41 patients who consumed other products, 35 consumed omega 3/6, 33 used probiotics, 12 consumed gemmotherapy and 11 consumed birch sap.

**Table 3:** The different natural health products consumed by patients

Natural health products	N	%
Vitamins	51	86.4%
Bach flowers & homeopathy	37	62.7%
Minerals	38	64.4%
Essential oils	38	64.4%
Herbs	50	84.7%
Other products	41	69.5%

**Fig. 3:**
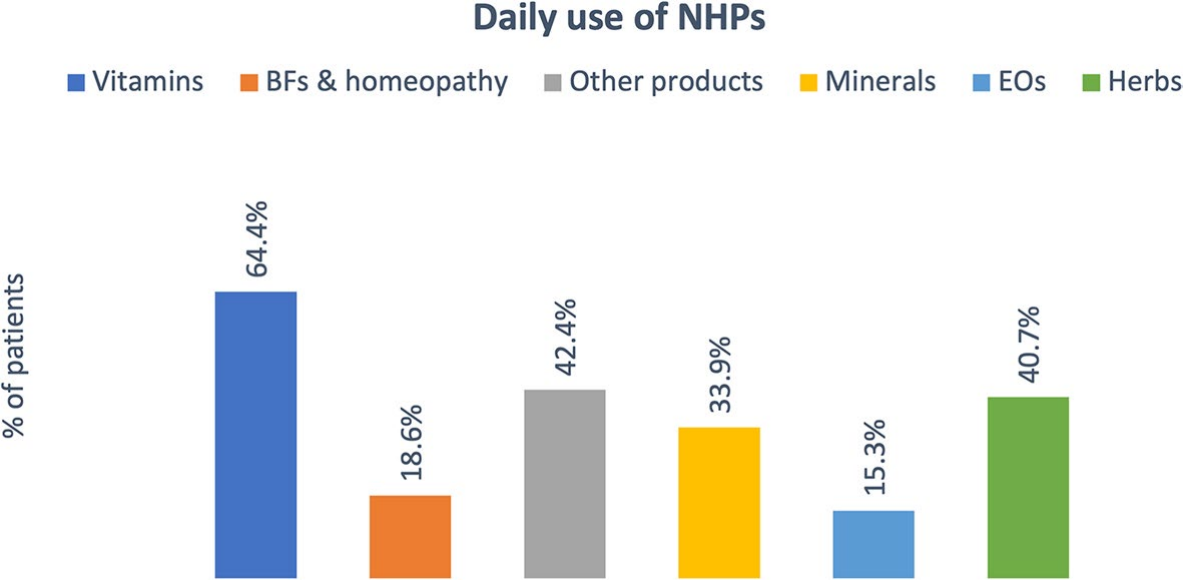
Daily use of NHPs

Details of daily consumption of these supplements are shown in Fig. 3.

**Fig. 4:**
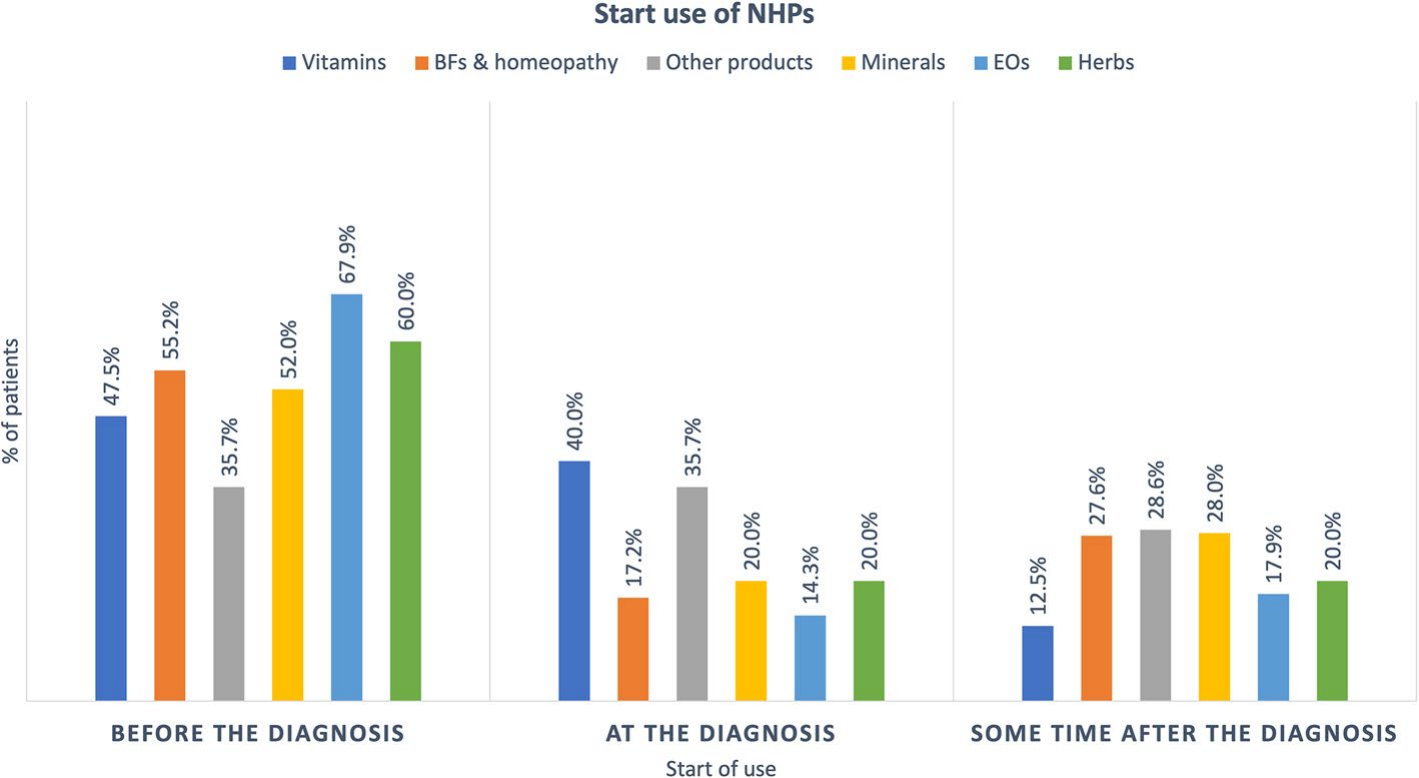
Start use of NHPs

Figure 4 illustrates that more than 50% of the patients consumed products from four of the six families prior to diagnosis. Vitamins and other products were the two exceptions. However, the $\chi ^{2}$ of independence did not show any statistical dependence between the NHPs type and the diagnosis. In contrast, 72.7 % (*n* = 24) of the patients reported a significant (*p* = 0.009) change in their NHPs consumption (in quantity and or frequency) since their diagnosis. Indeed, 33.3 % (*n* = 11) increased their consumption, 24.2 % (*n* = 8) changed their consumption by using other products and 15.2% (*n* = 5) reduced their consumption.

**Fig. 5:**
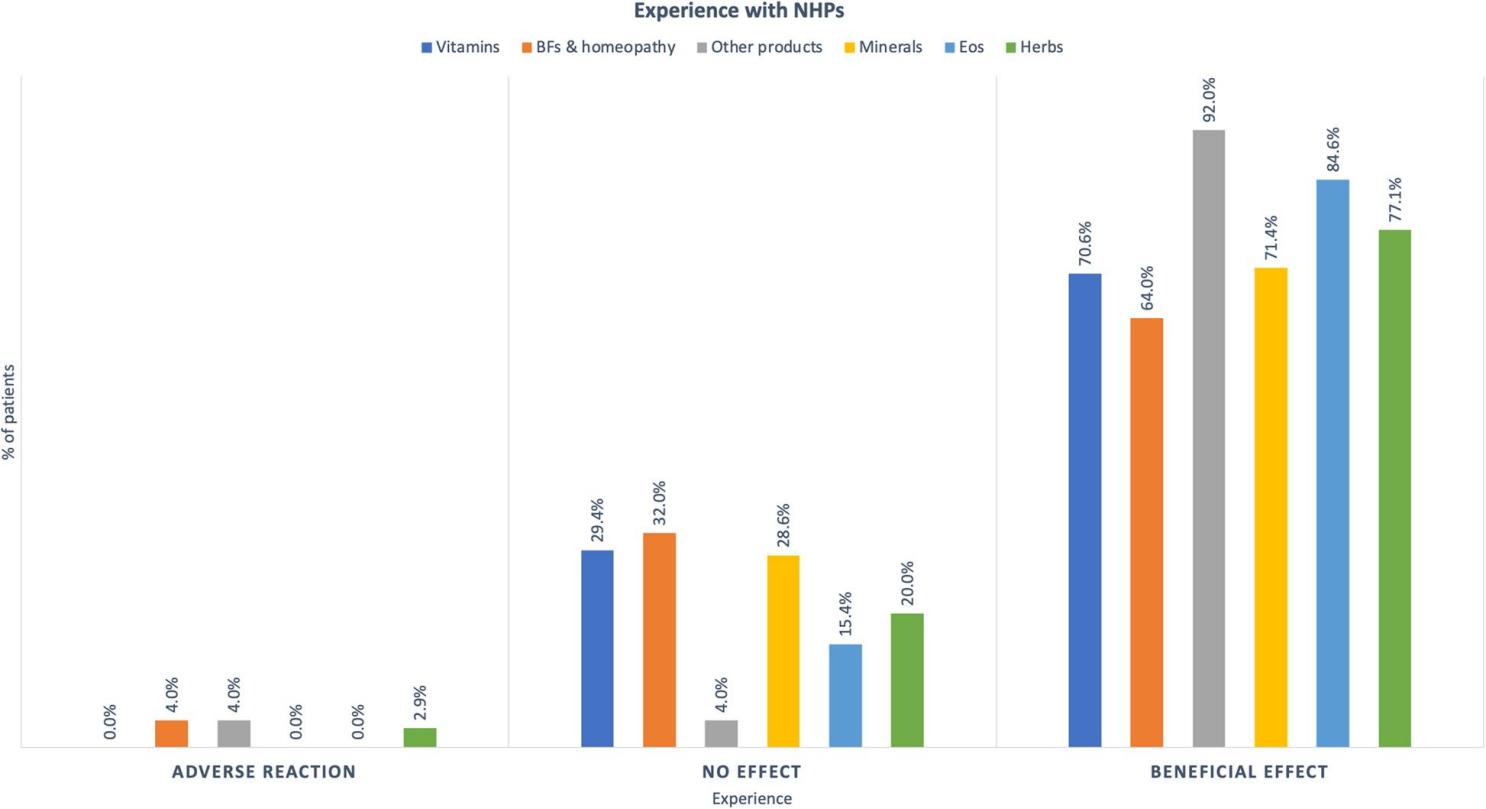
Experience with NHPs

Perceived benefits of complementary therapies were mostly reported by patients, regardless of the kind of supplements (Fig. 5). Adverse reactions were reported by three patients only. One patient reported ARs associated with homeopathy, another with probiotics, and the last with omega 3/6.

**Fig. 6:**
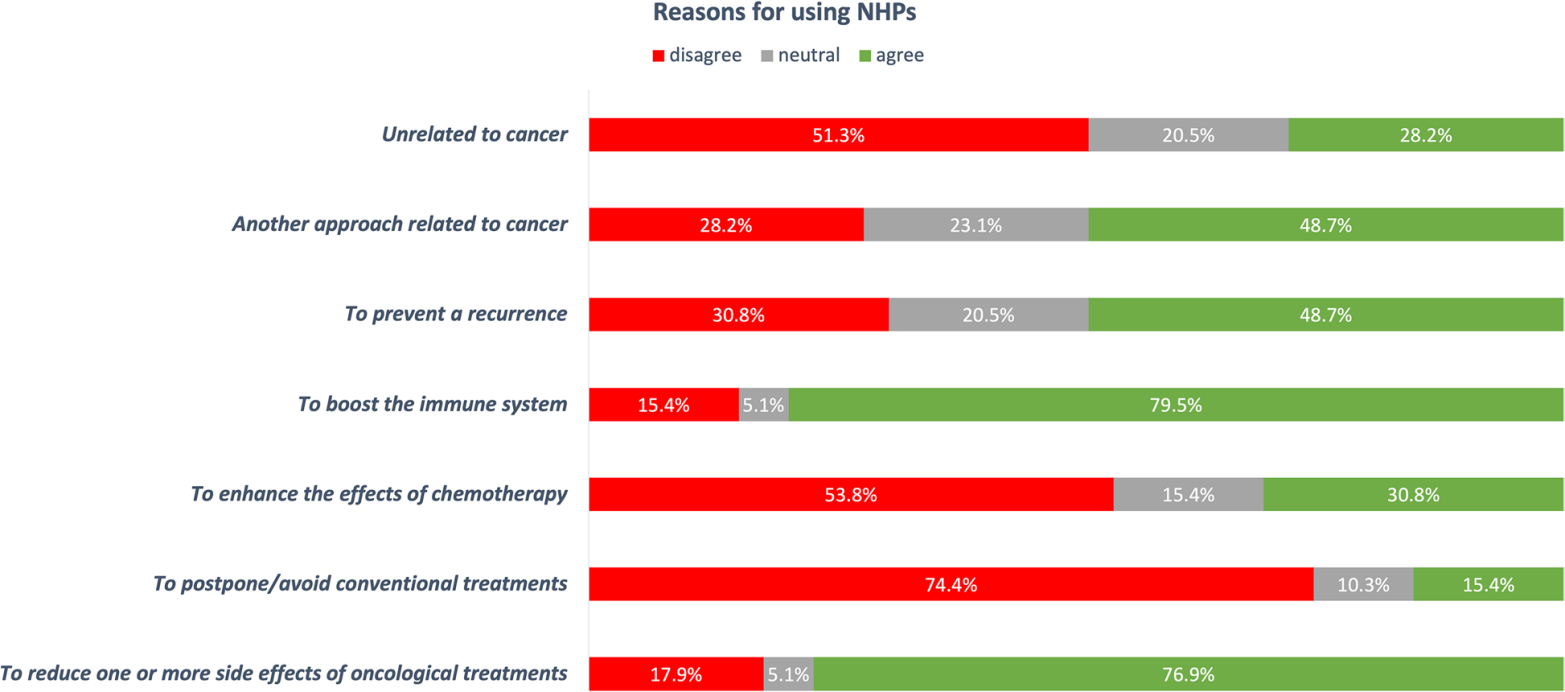
Reasons for using NHPs

Figure 6 illustrates the different reasons for NHPs’ use. On the one hand, patients mostly agreed with items “to boost the immune system” (79.7 %, *n* = 31) and “to reduce one or more side effects of cancer treatments” (76.9 %, *n* = 30). Almost half of the patients considered NHPs as another approach to cancer treatment that may also help to reduce recurrence. On the other hand, they mainly disagreed with the item “to postpone/avoid conventional treatment” (74.4 %, *n* = 24). Patients who did not use NHPs (*n* = 2) were asked to note why they did not do so. Price, lack of time to get the information and not knowing where to get the information were the reasons reported by the two patients who did not consume NHPs.

### Sources of information

**Fig. 7:**
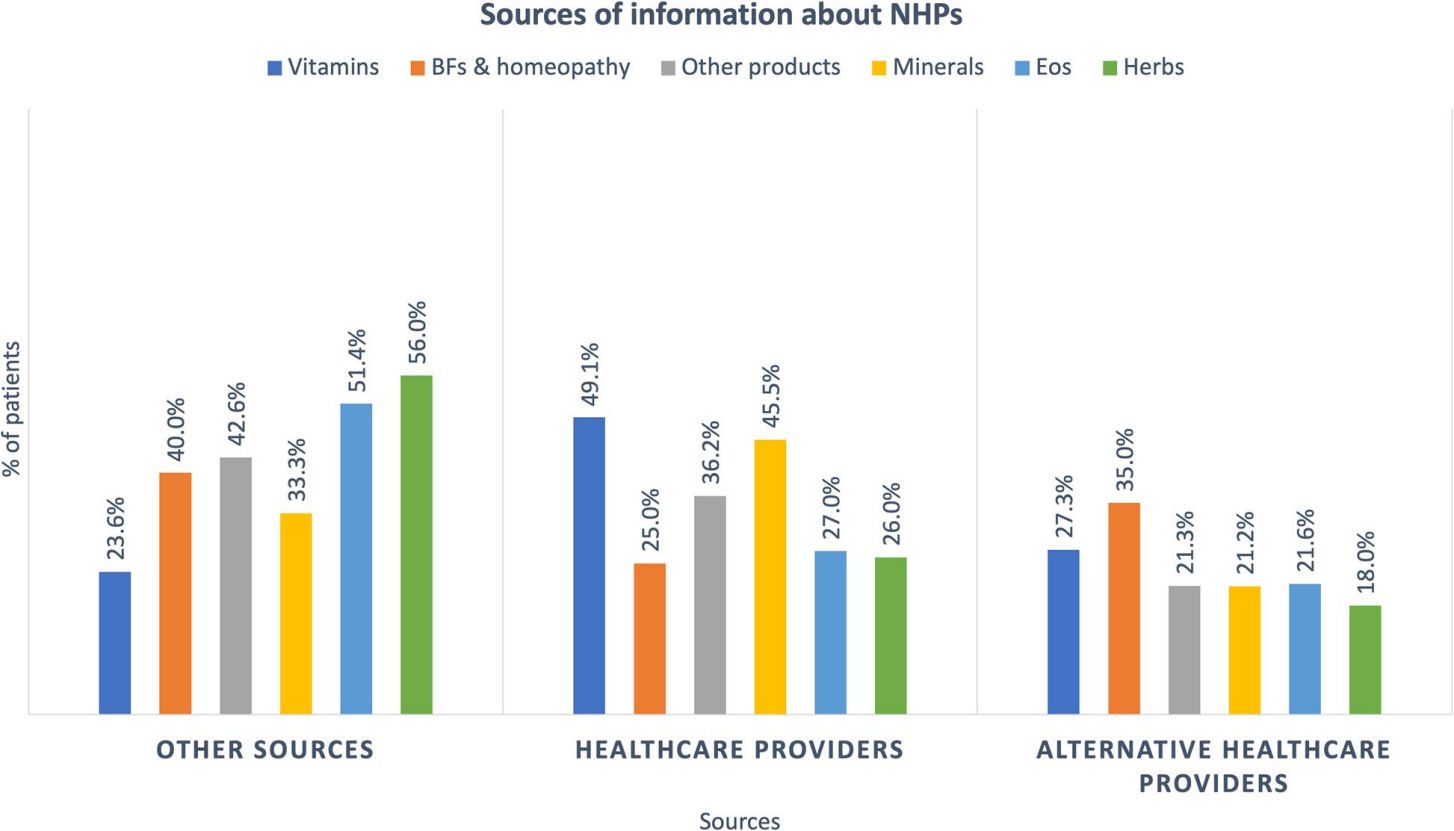
Sources of information

The use of BFs and homeopathy, other products, EOs and herbs derived mostly from other sources and less from medical and alternative-medical sources (i.e., homeopaths and naturopaths). Indeed, family, friends and their close circle were primarily reported as the source of information for BFs and homeopathy with 27.5 % (*n* = 14), other products with 26.2 % (*n* = 16), EOs with 36.6% (*n* = 15) and herbs with 25.4 % (*n* = 16). The homeopath was reported as the second leading source of information about homeopathy and BFs (23.5%). Vitamins and minerals, on the other hand, were prescribed mainly by HCPs. The two most essential vitamin information sources were the GP 23.8 % (*n* = 15) and the oncologist 20.6 % (*n* = 13). Figure 7 shows the distribution of the different sources for the six families.

## Discussion

This study describes the behaviors and attitudes of cancer patients toward natural complementary therapies in four hospitals in Belgium. The most popular products reported were vitamins and herbs, with 86.4 % and 84.7%, respectively. Other products were reported by 69.5 % of the patients. Studies found that vitamins, herbal products, and minerals are the most popular CAM supplements used by cancer patients [3, 6, 18, 43]. This contrasts with our results, where other products were consumed more than minerals. As discussed above, there is no consensus on what CAM is, making comparing results between studies complex. These studies do not mention whether probiotics, gemmotherapy, birch sap, and omega 3/6 were included. Currently, studies on gemmotherapy and birch sap use in cancer patients are lacking. On the other hand, probiotics and omega 3 use have increased significantly [44]. This is in line with our results, where we have three times more patients taking probiotics (*n* = 33) and omega 3/6 (*n* = 35) than birch sap (*n* = 11) and gemmotherapy (*n* = 12). Moreover, they seem pretty promising as complementary therapies in some cancer treatments. *In vitro* and *in vivo* studies on colorectal cancer models have proven the efficacy of probiotics’ antiproliferative and apoptotic benefits [45, 46]. Furthermore, omega 3 appear to have anticancer colorectal activity [47].

It has been shown previously that socioeconomic status, geography, and religious and spiritual beliefs influence the use of CAM [3, 7, 18]. Molassiotis et al. [6] found that herbal supplements were the most used therapy among all European cancer patients, but homeopathy was the CAM most used in Belgium. Homeopathy and BFs were the least used NHPs in our study. The different number of CAM users may explain this difference. Indeed, Molassiotis et al. [6] interviewed a sample size of 18 CAM users representative of Belgium, whereas we counted 57, about three times more. On the other hand, Saudi cancer patients are more likely to consume Zamzam water and camel products, such as milk or urine. This highlights how geography, religious and spiritual beliefs can influence CAM consumption [7, 36]. Moreover, being a woman under 60, having a higher education level and having a breast cancer diagnosis are predictors of CAM use [4, 6, 43].

Our results also show that 52.5 % of the patients consumed at least five different supplements. This high level of consumption highlights the importance of educating patients and HCPs about CAM-drug interactions risk [19]. In addition, we found that 33.3 % of the patients increased their consumption. Interestingly, the potential for CAM-drug interaction with cancer therapy was significantly associated with the number of CAM supplements taken. Even though all these supplements are at risk of interactions, natural substances can be classified into two categories.

On the one hand, some substances, such as antioxidants and herbal products, have a wide range of potential interactions. This makes them more likely to interact with conventional cancer therapy. For example, laboratory results showed that vitamin C can reduce the effects of chemotherapy drugs such as anthracyclines, bleomycin, bortezomib, and cisplatin. This is also the case for vitamin E whit tamoxifen antagonistic effects [24, 27]. In their study, Fasinu and Rapp [26] identified six herbal products - echinacea, garlic, ginseng, grapefruit juice, milk thistle, and St John’s wort - that have shown clinically relevant interactions with specific chemotherapeutic agents. For example, a patient on imatinib for seven years developed hepatotoxicity symptoms only three months after consuming an energy drink containing ginseng. When the ginseng-energy drinks were stopped, liver dysfunction disappeared. In addition to ginseng, energy drinks contain various compounds that may have contributed to liver dysfunction. In contrast to all these substances cited above, homeopathy and most minerals are natural substances unlikely to interact [27]. Furthermore, screening interactions for CAM-drug interactions through software is not as simple as identifying drug-drug interactions. Indeed, some programs only provide information on drug interactions, while others do not encompass all products on the wide NHPs market. This incongruence was highlighted by Végh et al. [48] among three different interactions databases.

Our survey found that 83.1 % of the patients believed complementary therapies could benefit CM. Our results also reveal that patients did not perceive complementary therapies as a threat to public health (89.9 %). Although, many are unaware that these non-mainstream medicines could interfere with their conventional treatment [3]. Our study found that most patients experienced beneficial effects and only a few reported adverse reactions. Borm et al. [49] reported similar results. It is also important to note that most patients cannot distinguish between dietary supplements and drugs due to their similar shapes, packaging, and trade names. Visual differences can be so slight that it is almost impossible to tell them apart. However, as mentioned in the introduction, the requirements for obtaining drug status are significantly higher than those for marketing authorization dietary supplements containing NHPs. Moreover, the European directive (2002/46/CE) [12] permits the free circulation of NHPs as dietary supplements, allowing patients access to a wide range of NHPs. Healthcare professionals should, therefore, educate patients about these products and make them aware of their potential risks [16, 17, 50].

McFadden et al. [40] investigated the relationship between attitudes toward CAM and their use in 65 healthy graduate students. They identified three factors influencing the use of CAM: a) possession of “philosophical congruence” with CAM, *which occurs when a patient identifies with (aspects of) the CAM modality’s cultures* [51] b) dissatisfaction with CM and c) holistic balance. A philosophical orientation congruent with CAM therapies was significantly correlated with present use (r=0.41, *p* = 0.001). A systematic review [38] showed that positive attitudes toward CAM and dissatisfaction with CM were the main reasons for CAM use among the general and condition-specific population. Our study focused on the nine items of philosophical congruence with CAM. According to our results, eight items seem to be consistent with those of McFadden et al. [40]. The only contradictory result is that 73.9 % of the patients did not believe complementary medicine is more effective than CM. This difference may be explained by our study involving cancer patients who were more aware that complementary therapies cannot replace conventional treatment. This observation also follow an Italian multi-survey led by Berreta et al. [5], which showed that almost all patients interviewed trust CM and oncological treatments. Most patients underlined the importance of considering a patient’s body, spirit, and mind when treating them. They also emphasized the importance of treating the whole person and integrating health beliefs and values into the healthcare process. This highlights the need for integrative oncology in the future.

A cancer diagnosis appears to be an essential factor that can influence the consumption of CAM. Indeed, the use of these products tends to increase at the time of diagnosis [5]. Buckner et al. [3] showed that biological products such as green tea, curcumin and ginger were consumed by 15 % of the patients before the diagnosis, while their consumption jumped up by 52 % of the patients after the diagnosis (*p* < 0,01). A European study [6] showed that CAM use was lower before diagnosis and increased by at least 30% after their diagnosis and that herbal medicines’ use tripled. Surprisingly, our study found that NHPs use was mostly initiated before the diagnosis, except for other products (i.e., probiotics, birch sap, gemmotherapy and omega 3/6). We conclude that the diagnosis did not affect the initiation of the type NHPs’ use, as demonstrated by the the $\chi ^{2}$ test of independence. Our results match those of Horneber et al. [21], who did not find a significant difference between the current and past use of CAM. This result can also be due to selection bias because the questionnaire naturally attracted more patients who use NHPs. However, 72.7 % of the patients reported significant changes in their consumption since their cancer diagnosis. In fact, we found that 15.2 % of the patients had reduced their consumption. This behavior change might have been explained by a discussion with their HCPs, who advised them to reduce their consumption of these products due to a lack of information about them. In 2012, a systematic review [52] reported that the prevalence of any Traditional, Complementary, and Alternative Medicine (TCAM) in the general population was up to 76 %. Moreover, the reported prevalence seemed to be underestimated. We found that most patients used NHPs before diagnosis, possibly due to increased use in the general population. Regardless of the initiation of CAM use, we showed also that the frequency of use varies over time. Therefore, HCPs must regularly inquire about their patients’ use of NHPs to detect any potential interactions [22].

We found that the primary source of information varies depending on the type of complementary therapies. On the one hand, media, family, friends, and their close circle were the most common sources for using of BFs and homeopathy, EOs, herbs and other products. These results align with previous studies [5, 6, 18]. Huebner et al. [39] demonstrated that 46 % of the patients trusted naturopaths and non-medical practitioners regarding CAM products. In our study, we observed that the homeopath was the second most influential source of information after family, friends, and the close circle for the consumption of homeopathy and BFs. Interestingly, a study [34] found that patients would like to have more information mainly from their oncologist. They also reported that complementary medicine users trusted the received information (82%), no matter the source, but almost all (73 %) admitted that additional information would be necessary. On the other hand, HCPs were the main source of information about vitamins and minerals. This is not surprising since these supplements (vitamins and minerals) are often prescribed or advised by doctors. We must highlight that our study did not distinguish prescribed from non-prescribed supplements. Nevertheless, compared to other studies [5, 6, 18, 34], we categorized sources of information according to the type of NHPs.

According to our survey, the main reasons for using NHPs shared by patients were to boost their immune system and to reduce one or more side effects of their oncological treatment. These results are consistent with several studies [20, 39, 53]. Keene et al. [10] used a method of grouping to identify cancer patients’ motivations for consuming CAM. Based on this systematic review, the most common reasons people sought CAM were to influence their cancer and to treat symptoms or side effects of their cancer. The primary reason for including “influence general health” in the category was to increase immunity. During the COVID-19 health crisis, there was a significant interest in some NHPs due to their perceived “immune-boosting” effects. This led to a surge in the consumption of various products, including vitamins C and D, zinc, omega 3, and herbal products such as garlic, turmeric, and ginger. Consequently, the daily intake of vitamins and herbs significantly increased during this period [54, 55]. In an era when immunotherapy is increasingly becoming a promising cancer therapeutic approach, probiotics, and omega 3 may be crucial to their efficacy thanks to their influence on the microbiome. Indeed, multiple studies [45, 46, 56, 57] have shown that the type of microbiome in a patient could be the main factor in the immunotherapy response. Furthermore, mistletoe may also enhance immunotherapy effectiveness through its immunomodulatory properties [56]. Identifying the reasons for using CAM could help HCPs discuss its use with their patients. Several studies [30, 31, 58] noted that one of the most common reasons for the nondisclosure of CAM use was only doctors not asking about CAM intake.

According to our findings, 74.4 % of the patients did not want to postpone/avoid conventional therapy. This result is consistent with integrative medicine, which implies a more patient-centered approach. Using these therapies makes patients feel that they have more control and are actively involved in their treatment [3, 27, 49].

One of the main strengths of our study is the distinction between the various types of NHPs and how patients consume (i.e., the source of information, the initiation time, and the way they experience NHP consumption) these NHPs. Identifying the type of substances consumed daily would make it possible to target these substances for potential drug-CAM interactions. Our study incorporated certain substances such as gemmotherapy and birch sap. To date there have been no studies of patients’ habits toward this type of substance. Despite the small numbers of patients consuming these substances, more studies are needed to eliminate any possible risks.

Furthermore, the fact that other substances, such as probiotics and omega 3, are promising potential adjuvants confirms the importance of integrative oncology. Indeed, despite the many risks these natural substances pose, they can be beneficial in some way. Knowing that diagnosis influences patients’ drug-taking behavior highlights the importance of healthcare professionals discussing their patients’ drug-taking habits with them. Identifying patients’ beliefs about CAM use benefit conventional medicine by incorporating some aspects of CAM into traditional medical treatments.

Despite this, our analysis contains some areas for improvement, including a low number of questionnaires due to many incomplete questionnaires (*n* = 237). As such, our study only reports the use and experiences of a small French-speaking cancer patients’ sample in Belgium. The COVID-19 global pandemic may be responsible for fewer visits to oncologists and hospital admissions for cancer patients. Angelini et al.’s systematic review and meta-analysis [59] found a significant decline in visits worldwide from January to October 2020 compared to pre-pandemic times. In Europe, overall visits for cancer patients decreased by 39.0% (-46.7; -31.3). Additionally, we investigated the use of complementary therapies alongside conventional treatments. Therefore, our results are not representative of the oncological population in Belgium. Further studies are necessary to investigate the use of CAM, experiences, and beliefs of cancer patients in a much larger sample, alongside CM but also instead of CM. Using the Likert scale can be both a strength and a weakness. The Likert scale provides more details about perceptions, opinions, and behaviors than binary questions and is quicker to fill out for respondents than open-ended questions. However, filling out questionnaires can be long, and patients may lose patience and choose their answers less attentively over time. Respondents may also avoid extreme items to appear more “normal”, referring to response bias. Because the translation from English to French, some questions may be interpreted differently by respondents, especially items regarding patients’ attitudes toward complementary therapies. Finally, using a self-completed questionnaire compared to interviews may be the reason for our low response rate, as suggested by Horneber et al. [21]. Moreover, using interviews would allow us to determine the exact consumption of patients and establish an objective classification based on assigned status according to legislation. However, a recent systematic review [10] showed that the difference in the prevalence of CAM use between self-completed questionnaires and face-to-face or telephone interviews was not statistically significant.

## Conclusion

Our results suggest that vitamins and herbs are the most widely consumed NHPs. Cancer patients who used NHPs show a positive attitude toward complementary therapies without trying to postpone or avoid conventional treatments. About one-half of patients consume at least five different NHPs. Therefore, this result emphasizes the importance that HCPs should be able to provide sufficient information about natural complementary therapies. Secondly, the consumption of complementary supplements should be systemically documented in patients’ records and interactions should be frequently checked by HCPs to avoid CAM-drug interactions [25]. Boosting the immune system and decreasing side effects related to conventional treatments are the main reasons for using complementary therapies. The source of information is variable and depends on the type of NHP consumed. Most of the patients started using NHPs before they were diagnosed. Further studies are required because of the discrepancy between our results and other studies. Furthermore, these therapies are becoming increasingly popular with the general population. For example, interviews could be another method to identify precisely which complementary therapy cancer patients use. This would enable us to better understand the behaviors of cancer patients in Belgium toward these complementary therapies. Knowing the patient’s reasons and understanding their attitudes toward complementary therapies will be useful for HCPs to address the topic with their patient [24]. The concept of integrative oncology could be a solution to ensure a better follow-up of the cancer patient, which brings conventional medicine and complementary approaches together in a coordinated way, establishing significant interactions between both [1].

### Supplementary Information

**Additional file 1.** Questionnaire_eng_Schils et al_110723.pdf; English translation of used French questionnaire.

## Data Availability

The data that support the findings of this study are available from C De Vriese and A Schils but restrictions apply to the availability of these data, which were used under license for the current study, and so are not publicly available. Data are however available from the authors upon reasonable request and with permission of C De Vriese.

## Abbreviations

ULB: Université Libre de Bruxelles
NHPs: Natural health products
CAM: Complementary and alternative medicine
CM: Conventional medicine
HCPs: Healthcare providers
NCCIH: National Center for Complementary and Integrative Health
ARs: Adverse reactions
BFs: Bach flowers
Eos: Essential oils
CACMAS: Complementary and Alternative Conventional Medicine Scale
CHR: Centre Hospitalier Régional
GPs: General practitioners
