# Supplementary material for: Cancer patients’ behaviors and attitudes toward natural health products
Source: BMC Complement Med Ther. 2023 Dec 6;23:442. doi: 10.1186/s12906-023-04278-0 (PMC10698981; doi:10.1186/s12906-023-04278-0)
Supplement: Supplementary file 1 — Additional file 1. Questionnaire_eng_Schils et al_110723.pdf; English translation of used French questionnaire. [file 12906_2023_4278_MOESM1_ESM.pdf]

Contribution to the study of cancer patients' natural health products consumption

Complementary medicines online questionnaire

Hello,

This questionnaire is part of a study of the perception and possible use of complementary natural therapies by patients suffering from cancer or in remission. The main aim of the study is to find out more about the use of complementary therapies by patients undergoing chemotherapy (injected or oral) or who have undergone chemotherapy in the last 5 years, and to understand the reasons why they use them.

This study will form the basis of a multidisciplinary research project.

It will take you around ten minutes to complete this questionnaire. We guarantee anonymity regarding your answers to this questionnaire, as provided for in the European General Regulation on Personal Data Protection of 25 May 2018. We insist that no judgement will be made on your answers. We invite you to answer as honestly as possible.

Thank you in advance for your participation and time to this project.

1.Are you the patient?

- o Yes
- o No

If you are not the patient, complete the questionnaire in his or her presence to ensure the accuracy of the answers you give.

2.In which year were you diagnosed with your cancer(s)?

3.Have you received one or more anti-cancer drugs and/or hormone therapy through injection or orally?

- o Yes
- o No

4.Do you know the name of anti-cancer or hormone therapy drug(s) that you have received, whether through injection or orally?
If yes, which ones?

For cancer treatment, you are or have been under the care of an oncologist in conventional medicine. However, other therapists may also be involved in your care such as therapists who have a different approach to conventional medicine: naturopaths, homeopaths, doctors specializing in phytotherapy.

To find out how you view complementary therapies (herbal teas, plant-based products, vitamins, minerals, essential oils, etc.), please rate the following from 0 (strongly disagree) to 10 (strongly agree).

	1	2	3	4	5	6	7	8	9	10
**5. The health of my body, mind, and spirit are related, and whoever cares for my health should take them into account.**	○	○	○	○	○	○	○	○	○	○
**6. Effects of complementary therapies are usually the result of a placebo effect.**	○	○	○	○	○	○	○	○	○	○
**7. Complementary therapies are a threat to public health.**	○	○	○	○	○	○	○	○	○	○
**8. I believe that complementary medicine enables me to take a more active part in maintaining my health.**	○	○	○	○	○	○	○	○	○	○
**9. Complementary therapies include ideas and methods from which conventional medicine could benefit.**	○	○	○	○	○	○	○	○	○	○
**10. Treatments not tested in a scientifically recognized manner should be discouraged.**	○	○	○	○	○	○	○	○	○	○
**11. I believe that complementary therapy will be more effective for my problem than orthodox medicine.**	○	○	○	○	○	○	○	○	○	○
**12. I value the emphasis on treating the whole person.**	○	○	○	○	○	○	○	○	○	○
**13. A patient's expectations, health beliefs, and values should be integrated into the health care process.**	○	○	○	○	○	○	○	○	○	○

14.What complementary natural therapies do you or have you used, and how often? (several answers possible)
Capsules/drops/granules/tablets of:

	**Daily**	**Weekly**	**Monthly**	**Less than once a month**
**Vitamins**	○	○	○	○
**Bach flowers**	○	○	○	○
**Omega 3/6**	○	○	○	○
**Homeopathy**	○	○	○	○
**Minerals**	○	○	○	○
**Probiotics**	○	○	○	○
**Gemmotherapy**	○	○	○	○
**Birch sap**	○	○	○	○
**Essential oils**	○	○	○	○
**Plants**	○	○	○	○
**Herbal teas**	○	○	○	○

All the product types listed in the previous tables will be referred to as “natural health products” in the rest of the questionnaire.

16+17
How long have you used this natural health product (vitamins)?
- I used it before my cancer diagnosis.
- I have used it since my diagnosis.
- I have used it for some time after my diagnosis.

Vitamins: How did you choose this type of natural health product or who recommended it to you?
- The media (internet/TV/radio)
- My close circle (family/friends/acquaintances/patient groups)
- My nurse
- My oncologist
- My homeopath
- My pharmacist
- My naturopath
- My physiotherapist
- My general practitioner

How long have you used this natural health product (Bach flowers)?
- I used it before my cancer diagnosis.
- I have used it since my diagnosis.
- I have used it for some time after my diagnosis.

Bach flowers: How did you choose this type of natural health product or who recommended it to you?
- The media (internet/TV/radio)
- My close circle (family/friends/acquaintances/patient groups)
- My nurse
- My oncologist
- My homeopath
- My pharmacist
- My naturopath
- My physiotherapist
- My general practitioner

How long have you used this natural health product (Omega 3/6)?
- I used it before my cancer diagnosis.
- I have used it since my diagnosis.
- I have used it for some time after my diagnosis.

Omega 3/6: How did you choose this type of natural health product or who recommended it to you?

- The media (internet/TV/radio)
- My close circle (family/friends/acquaintances/patient groups)
- My nurse
- My oncologist
- My homeopath
- My pharmacist
- My naturopath
- My physiotherapist
- My general practitioner

How long have you used this natural health product (homeopathy)?

- I used it before my cancer diagnosis.
- I have used it since my diagnosis.
- I have used it for some time after my diagnosis.

Homeopathy: How did you choose this type of natural health product or who recommended it to you?

- The media (internet/TV/radio)
- My close circle (family/friends/acquaintances/patient groups)
- My nurse
- My oncologist
- My homeopath
- My pharmacist
- My naturopath
- My physiotherapist
- My general practitioner

How long have you used this natural health product (minerals)?

- I used it before my cancer diagnosis.
- I have used it since my diagnosis.
- I have used it for some time after my diagnosis.

Minerals: How did you choose this type of natural health product or who recommended it to you?

- The media (internet/TV/radio)
- My close circle (family/friends/acquaintances/patient groups)
- My nurse
- My oncologist
- My homeopath
- My pharmacist
- My naturopath
- My physiotherapist

- My general practitioner

How long have you used this natural health product (probiotics)?

- I used it before my cancer diagnosis.
- I have used it since my diagnosis.
- I have used it for some time after my diagnosis.

Probiotics: How did you choose this type of natural health product or who recommended it to you?

- The media (internet/TV/radio)
- My close circle (family/friends/acquaintances/patient groups)
- My nurse
- My oncologist
- My homeopath
- My pharmacist
- My naturopath
- My physiotherapist
- My general practitioner

How long have you used this natural health product (gemmotherapy)?

- I used it before my cancer diagnosis.
- I have used it since my diagnosis.
- I have used it for some time after my diagnosis.

Gemmotherapy: How did you choose this type of natural health product or who recommended it to you?

- The media (internet/TV/radio)
- My close circle (family/friends/acquaintances/patient groups)
- My nurse
- My oncologist
- My homeopath
- My pharmacist
- My naturopath
- My physiotherapist
- My general practitioner

How long have you used this natural health product (birch sap)?

- I used it before my cancer diagnosis.
- I have used it since my diagnosis.
- I have used it for some time after my diagnosis.

Birch sap: How did you choose this type of natural health product or who recommended it to you?

- The media (internet/TV/radio)
- My close circle (family/friends/acquaintances/patient groups)
- My nurse
- My oncologist
- My homeopath
- My pharmacist
- My naturopath
- My physiotherapist
- My general practitioner

How long have you used this natural health product (essential oils)?

- I used it before my cancer diagnosis.
- I have used it since my diagnosis.
- I have used it for some time after my diagnosis.

Essential oils: How did you choose this type of natural health product or who recommended it to you?

- The media (internet/TV/radio)
- My close circle (family/friends/acquaintances/patient groups)
- My nurse
- My oncologist
- My homeopath
- My pharmacist
- My naturopath
- My physiotherapist
- My general practitioner

How long have you used this natural health product (plants)?

- I used it before my cancer diagnosis.
- I have used it since my diagnosis.
- I have used it for some time after my diagnosis.

Plants: How did you choose this type of natural health product or who recommended it to you?

- The media (internet/TV/radio)
- My close circle (family/friends/acquaintances/patient groups)
- My nurse
- My oncologist
- My homeopath
- My pharmacist
- My naturopath

- My physiotherapist
- My general practitioner

How long have you used this natural health product (herbal teas)?

- I used it before my cancer diagnosis.
- I have used it since my diagnosis.
- I have used it for some time after my diagnosis.

Herbal teas: How did you choose this type of natural health product or who recommended it to you?

- The media (internet/TV/radio)
- My close circle (family/friends/acquaintances/patient groups)
- My nurse
- My oncologist
- My homeopath
- My pharmacist
- My naturopath
- My physiotherapist
- My general practitioner

17.What is you experience of these natural health products?

	I felt the beneficial effects	I have experienced adverse effects	I felt no effect
**Vitamins**	○	○	○
**Bach flowers**	○	○	○
**Omega 3/6**	○	○	○
**Homeopathy**	○	○	○
**Minerals**	○	○	○
**Probiotics**	○	○	○
**Gemmotherapy**	○	○	○
**Birch sap**	○	○	○
**Essential oils**	○	○	○
**Plants**	○	○	○
**Herbal teas**	○	○	○

18.If you used natural health products, have you changed your use since your cancer diagnosis?

- My consumption has not changed.

- I use more (in quantity and/or frequency).
- I use less (in quantity and/or frequency).
- I am currently using different products compared to what I used in the past.

19.Why have you used and/or do you use natural health products? Rate the following reasons from strongly disagree to strongly agree.

	Strongly disagree	Disagree	Neutral	Agree	Strongly agree
I want/wanted to reduce one or more side effects of my oncological treatments.	○	○	○	○	○
I want/wanted to postpone/avoid conventional treatments.	○	○	○	○	○
I want/wanted to enhance the effects of chemotherapy (complementarity).	○	○	○	○	○
I want/wanted to boost my immunity.	○	○	○	○	○
I want/wanted to prevent a recurrence.	○	○	○	○	○
I want/wanted another approach related to cancer.	○	○	○	○	○
My consumption is not related to my cancer.	○	○	○	○	○

20.Have you ever thought of using a natural health product? (only shown if patient has never consumed natural health product)

- Yes
- No

21.What prevented you from using complementary therapies? (several answers possible) (only shown if patient has never consumed natural health product)

- I didn't research it.
- I don't know where to find information.
- I have no time to waste.
- I don't believe in this type of therapy.
- It's too expensive.
- A health professional talked me out of it.
- Another person talked me out of it.
- Another reason.

22.You (patient) were born between:

- 1920-1929
- 1930-1939
- 1940-1949
- 1950-1959
- 1960-1969
- 1970-1979
- 1980-1989
- 1990-1999
- 2000-2001

23. You (patient) are:

- o Man
- o Women

24. In which country are you/were you treated for your cancer?

- o Belgium
- o France
- o Luxembourg
- o Switzerland
- o Other
